# Factors Associated with Seasonal Influenza Non-Vaccination Among Children with Chronic Health Conditions in Canada: A Cross-Sectional Study

**DOI:** 10.3390/vaccines14050396

**Published:** 2026-04-29

**Authors:** Arlanna Pugh, Sailly Dave, Marwa Ebrahim, Julie A. Laroche

**Affiliations:** Vaccine Coverage and Effectiveness Surveillance Division, Centre for Immunization surveillance and Programs, Infectious Diseases and Vaccination Programs Branch, Public Health Agency of Canada, Ottawa, ON K1A 0K9, Canada

**Keywords:** influenza vaccine, vaccine hesitancy, unvaccinated, chronic disease, children, parents

## Abstract

**Background/Objectives**: Seasonal influenza vaccination is pivotal for protecting high-risk populations, including those with chronic health conditions (CHCs), from severe complications and outcomes. This study aims to describe the sociodemographic characteristics of unvaccinated children (6 months–17 years old) with CHCs, the reasons why vaccine hesitant parents chose not to vaccinate their children, and the factors associated with seasonal influenza non-vaccination among these children. **Methods**: This cross-sectional analysis used a sub-sample from the 2023 Childhood COVID-19 Immunization Coverage Survey, which captured data between April and July 2023. Parent and child characteristics were explored, using frequencies and proportions. Weighted unadjusted, partially adjusted (with child age and sex at birth), and fully adjusted multivariable quasi-Poisson models were designed to identify socio-demographic factors associated with seasonal influenza non-vaccination. **Results**: A total of 649/1187 (55%) children with CHCs were unvaccinated against influenza during the 2022–2023 influenza season. Vaccine hesitant parents with unvaccinated children expressed concerns with vaccine effectiveness (39.1%) and safety (27.5%), as did parents who refused vaccination for their child (56.1% and 35.8%, respectively). Unvaccinated parents (RR: 4.549, 99% CI: 4.480, 4.619) and parents with low household income (RR: 1.428, 99% CI: 1.400, 1.456) were more likely to have unvaccinated children, whereas children who received an annual influenza vaccine (RR: 0.097, 99% CI: 0.094, 0.100) and did not have a disability (RR: 0.913, 99% CI: 0.904, 0.922) had a lower likelihood of non-vaccination. **Conclusions**: These findings highlight the need for renewed messaging and educational resources targeting vaccine hesitancy and misinformation prevalent among parents with vulnerable youth.

## 1. Introduction

Seasonal influenza, a highly contagious respiratory viral infection, is a significant contributor to global morbidity and mortality each year [1]. It is estimated to infect up to 1 billion people world-wide and is associated with approximately 290,000 to 650,000 deaths annually [2]. While most cases of influenza are mild among healthy individuals, those who are immunocompromised, who have chronic health conditions (CHCs) (e.g., cancer, diabetes, cardiac disease), or who are over 65 years of age or under 5 years of age, are at greater risk of severe complications, hospitalization, or death [1].

Children are at a higher risk of influenza-related complications such as secondary bacterial infections (e.g., pneumonia), ear infections, and in rare cases, neurologic, cardiovascular or musculoskeletal complications [3]. It has also been shown that children ≤ 10 years old with chronic lung diseases, such as asthma and cystic fibrosis, are about five times more likely to be hospitalized due to influenza infection than those without chronic lung diseases [4]. Once infected, they can effectively spread the virus to other vulnerable populations through close contact in school and home settings, due to extended periods of viral shedding after illness [5,6]. Such transmission dynamics can have measurable public health consequences at the population level.

In Canada, the 2022–2023 influenza season was marked by a significant resurgence of influenza activity since the start of the COVID-19 pandemic and had a tremendous effect on children and youth. Approximately 45% (n = 6194/13,729) of reported influenza A(H3N2) cases were in the pediatric population (less than 19 years of age), compared to an average of 17% in pre-pandemic seasons [7]. Furthermore, weekly influenza-related hospitalizations among this population were consistently above historical peaks for several weeks. In total, influenza-related hospitalizations surpassed historical averages during the 2022–2023 season [7]. Given the negative consequences of influenza and the impact it has on children, preventative measures are of utmost importance to protect those at risk.

Vaccination remains the primary strategy to mitigate the impacts of seasonal influenza infection. The Canadian National Advisory Committee on Immunization recommends all individuals ≥ 6 months of age who are without contraindications receive an annual influenza vaccine, particularly those at high-risk of influenza-related complications [8]. Vaccination among youth has been shown in some cases to curb transmission and protect those in the community who may be at high-risk of severe disease [9,10]. In Canada, trivalent and quadrivalent standard-dose influenza vaccines (among others) are available to children 2 to 17 years old with CHCs [8]. However, despite consistent messaging encouraging annual vaccination among high-risk populations, vaccine coverage among children with CHCs remains low (36%) [11]. This is corroborated by other studies, which similarly report influenza vaccination coverage of approximately one-third among children with CHCs [12,13,14].

Global efforts have been made to better understand poor vaccine uptake among high-risk populations, along with factors associated with vaccine hesitancy especially among children with CHCs. One systematic review exploring influenza vaccine hesitancy among high-risk populations predominantly from American and European regions, found that individuals with CHCs most frequently cited barriers to vaccine uptake when they lacked confidence in the vaccine or were complacent towards receiving it [15]. Gaining insights into drivers or barriers of influenza vaccination in children with CHCs will allow for tailored public health interventions and support to help parents with decision-making.

To date, seasonal influenza vaccine coverage among children with CHCs in Canada remains poorly characterized, as does our understanding of factors associated with non-vaccination in this cohort. This study aims to address these gaps by examining the sociodemographic characteristics of unvaccinated children with CHCs and their parents, exploring parental reasons for seasonal influenza non-vaccination, vaccine hesitancy or refusal, and identifying sociodemographic and vaccine-related factors associated with seasonal influenza non-vaccination among children with CHCs.

## 2. Materials and Methods

### 2.1. Data Source

This study uses data from the 2023 Childhood COVID-19 Immunization Coverage Survey (CCICS), a nationally representative, cross-sectional survey of parents and guardians (hereafter referred to as parents), residing in all Canadian provinces and territories with children under 18 years of age. Data were collected between 11 April 2023, and 26 July 2023, towards the end of the 2022–2023 seasonal influenza season. It captured information on COVID-19 and seasonal influenza immunization coverage among children, parental intentions to vaccinate their children, and parental knowledge, attitudes, and beliefs about these vaccines.

The survey employed a multi-modal approach using random digit dialling to recruit participants and either online survey or computer-assisted telephone interviews to administer the survey. To enhance sample representativeness, sampling quotas were implemented for child age group, child sex (at birth), and province/territory of residence. Post-stratification (direct) sampling weights based on these three variables were created using population totals from the Statistics Canada 2021 Census. These weights were applied when calculating population estimates for this survey. This method was also used, along with drawing random samples with replacement from the primary sampling units, to calculate 500 mean bootstrap weights. These bootstrap weights were then used to calculate population-based confidence intervals. The survey captured a total of 11,395 responses, of which 51.1% of children were male and 48.9% were female.

The CCICS 2023 was approved by the Health Canada and Public Health Agency of Canada Research Ethics Board. Additional details on survey methodology have been previously documented, such as survey participation and handling non-response bias [16].

### 2.2. Analytic Sample

**Figure 1:**
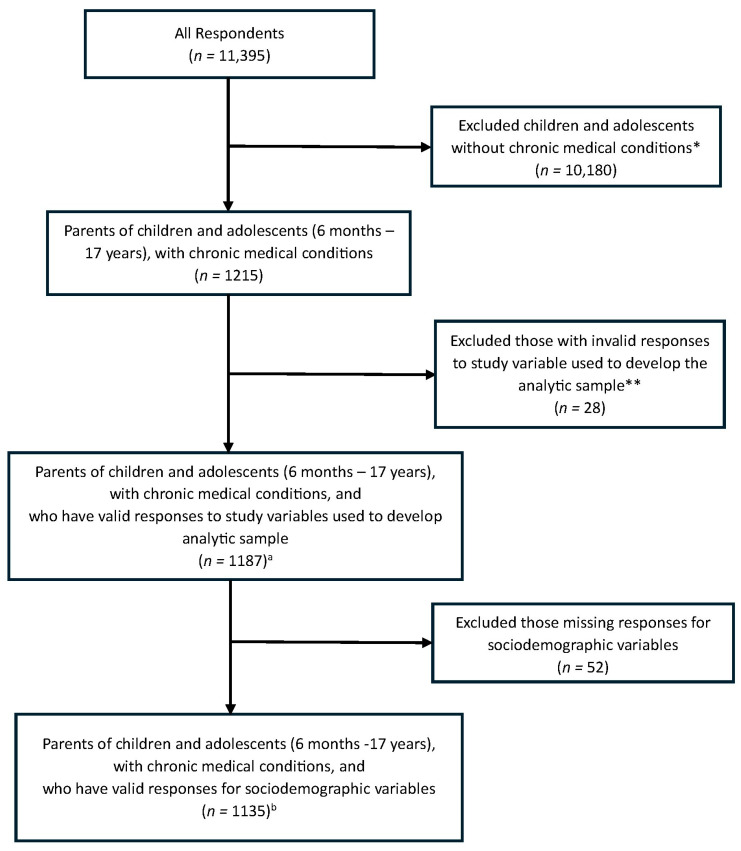
Analytical Sample. ^a^ Analytic sample for descriptive statistics. ^b^ Analytic sample for multivariate quasi-Poisson regression. * Excluded those who responded “No”, “Don’t Know”, “Prefer Not to Answer” to the following statement: “Does your child have any of the following [chronic medical] conditions?” ** “Don’t Know”, “Prefer Not to Answer”, and/or “Skipped” to the following statements: “Did [child’s nickname] receive a flu vaccine this flu season, between September 2022 and March 2023?” (n = 28).

Parents were asked “Does your child have any of the following conditions?” and were provided a list of conditions to select any that apply (based on parental self-report): sickle cell anemia or thalassemia major; neurologic or neurodevelopmental disorders; asthma or other chronic lung disease; chronic liver, heart or kidney disease; diabetes, obesity or Down syndrome; immune suppression; cancer; and other medical condition, which refers to endocrine and autoimmune disorders [17]. Those who selected at least one condition were included in this secondary data analysis. Parents who had invalid or missing responses (i.e., don’t know or prefer not to answer) for the child seasonal influenza vaccination status variable were also removed from the analytic sample. This resulted in a total sample of 1187 parents for descriptive analysis (Figure 1).

We also examined parental reasons for hesitancy and refusal among children who were not vaccinated. Parents who were hesitant or refused to vaccinate their child against seasonal influenza were identified by the following survey questions: “Were you reluctant or hesitant to vaccinate your child against the flu during this flu season?” and “Did you refuse to get the flu vaccine for your child during this flu season?” Reasons for hesitancy or refusal among parents who answered “Yes” to these questions were summarized and ranked, using frequencies and weighted proportions.

For multivariable regression analyses, a complete case analysis was considered for all sociodemographic and vaccine-specific variables of interest. Several studies have suggested there is a greater likelihood of bias in study results and a decrease in statistical power when more than 10% of cases have missing data [18,19]. To be more conservative in our assessment of missingness, patterns of missingness were explored for variables that were missing more than 5% of cases [20]. If patterns were identified, missing cases would subsequently be coded into a distinct “Missing” category for that variable. Variables with a “Missing” category included: household income, parent educational status, childhood annual flu vaccination, and parent reluctant or hesitant to vaccinate child against flu this flu season. Once variables were recoded, a total of 52 cases remained missing (4.4% of descriptive sample). This proportion of missing cases was considered acceptable; therefore, these cases were removed from the analytic sample. A total of 1135 parents were included in the final analytic sample for multivariable regression analysis (Figure 1).

### 2.3. Study Variables

Seasonal influenza vaccination status of the child was the dependent variable of interest for this study. Parents who provided a “Yes” or “No” response to the survey question: “Did [child’s nickname] receive a flu vaccine this flu season, between September 2022 and March 2023?” were included in this binary outcome. A variety of sociodemographic variables were explored, including parent and child age, sex, and ethnicity; household income and setting; parent employment status and education; parent citizenship status; and the presence of child asthma and disability. Asthma status was selected for further analysis as an independent variable given its well-documented role as a comorbidity associated with seasonal influenza-related hospitalization and clinical outcomes [21,22,23]. Vaccine-specific variables were also explored, such as parent and child annual flu vaccination and past flu vaccination; parental reluctance or hesitancy to vaccinate themselves or their children this flu season; parental intention to vaccinate their children next flu season; and routine childhood vaccinations. Categories for some variables were merged to facilitate analysis and ensure adequate cell counts.

### 2.4. Data Analysis

Unweighted frequencies and weighted percentages were computed for all sociodemographic and vaccine-specific variables of interest independently and by child seasonal influenza vaccination status. To determine statistical significance between variable groups, we used the Pearson’s chi-square test (or Fisher’s exact test when expected cell counts were 5 or less). To calculate robust 95% Wald confidence intervals for weighted estimates, a total of 500 mean bootstrap weights were applied.

Given that our outcome of interest is common in our sample (i.e., greater than 10%), we used quasi-Poisson regression with a log link to model our binary outcome and estimate prevalence ratios directly [24,25,26,27]. Model variance was estimated using 500 mean bootstrap weights which, under this approach, is derived from the spread of coefficient estimates across bootstrap resamples rather than from the standard Poisson variance function [28]. This reflects the complex survey design of the CCICS and ensures that confidence intervals for the models are calculated correctly.

The first set of models were unadjusted and produced crude risk ratios for each variable of interest and the second set were adjusted for child age and sex (at birth). Given that our research objectives are descriptive in nature (and not causal or predictive), our second set of models quantify associations between our outcome and variables of interest individually, while also controlling for age and sex (at birth). This allows for a simpler interpretation of associations without inadvertently introducing effect mediation and/or collider bias when additional variables are considered [29].

The third set featured one model that was adjusted for child age and sex (at birth), and considered including additional covariates shown in the literature to be of public health interest or potentially correlated with the outcome, such as household income, urban/rural setting, employment and education status, ethnicity, parental hesitancy, intention to vaccinate child in the next seasonal influenza season, uptake of routine vaccination, child’s asthma and disability status. Variables were selected for the final model by assessing survey-weighted variance inflation factors (VIF). Variables that were found to be highly correlated (i.e., VIF of 5 or greater) with others already included in the model were removed to avoid redundancy and unstable coefficient estimation. Multicollinearity was particularly common among parent- and child- level versions of conceptually similar variables (e.g., parent/child age, sex, ethnicity, annual flu vaccination, etc.), and in these cases, the child variables were retained in the model. All VIF values in the model were below 5, indicating acceptable levels of collinearity among retained factors. Model fit was assessed using survey-weighted area under the Receiver Operating Characteristic (ROC) curve values. While 95% confidence intervals (CIs) were used for the descriptive analyses, all three models used 99% CIs to provide more conservative estimates of precision given the exploratory nature of this analysis. Data analysis was conducted with R Statistical Software, version 4.3.2.

## 3. Results

### 3.1. Sociodemographic Characteristics of Unvaccinated Children and Their Parents

Of the 1187 parents of children with CHCs retained for descriptive analysis, 649 (54.7%) declared that their child was not vaccinated against seasonal influenza during the 2022–2023 season. Most parents with unvaccinated children were 40–59 years old (65.4%), assigned female sex at birth (69.6%), were employed (86.9%), lived in urban settings (83.7%), and were Canadian citizens by birth (81.3%). Similar demographic trends were shown among parents with vaccinated children, with a few notable exceptions. There were a greater proportion of parents with unvaccinated children who did not receive the flu vaccine themselves (82.1% vs. 9.0%), who were reluctant or hesitant to vaccinate themselves against the flu (38.8% vs. 4.4%), and who never got the flu vaccine annually (38.1% vs. 4.9%), compared to parents with vaccinated children (Table 1).

Among unvaccinated children, 14.8% were 6 months–4 years old, 42.1% were 5–11 years old, and 43.1% were 12–17 years old. Most unvaccinated children were assigned male sex at birth (56.9%), were of non-visible minority (68.3%), had asthma (55.2%), and received all their routine childhood vaccinations (86.2%). With regard to flu vaccination, the majority of unvaccinated children never received the flu vaccine annually (49.1%), had parents who were not vaccine hesitant/reluctant towards vaccinating their child during the 2022–2023 season (55.1%), and had parents who definitely or probably won’t vaccinate their child during the 2023–2024 season (54.6%) (Table 2).

### 3.2. Parental Reasons for Not Vaccinating Children Against Seasonal Influenza

Of the 262 parents with unvaccinated children that were hesitant or reluctant to vaccinate, 39.1% were concerned about vaccine effectiveness, 27.5% were concerned about vaccine safety, and 25.3% stated that (in their opinion) their child is not at risk of getting the flu or at risk of severe infection. Less than 5% of parents in the group selected misinformation, not knowing where to get reliable information, wanting to discuss the flu vaccine with their child’s health care practitioner, and being against vaccination as reasons for non-vaccination (Table 3).

When exploring reasons for non-vaccination among the 229 parents who refused the seasonal influenza vaccine for their child, 56.1% reported not considering it necessary, 35.8% reported concerns about the safety/side effects of the flu vaccine, and 24.9% reported (in their opinion) that the flu vaccine does not work. There was a similar proportion of parents who refused vaccination for their child due to religious or philosophical reasons (5.0%) and parents who were hesitant or reluctant to vaccinate (4.7%) (Table 4).

### 3.3. Determinants of Seasonal Influenza Non-Vaccination Among Children with CHCs

Risk ratio coefficients from the age and sex (at birth) adjusted quasi-Poisson models are presented in Table 5, and suggest that parents who are 40 to 59 years old (compared to 18 to 39 years old), who are unemployed (compared to those employed), and who have at least a bachelor’s degree (compared to completing at least a high school diploma) are less likely to have children unvaccinated against seasonal influenza. When exploring vaccination behaviours and perspectives, there is a clear gradient in decreasing likelihood of having unvaccinated children shown for parents who receive the seasonal influenza vaccine some flu seasons (RR: 0.825, 99% CI: 0.818, 0.833), most flu seasons (RR: 0.429, 99% CI: 0.423, 0.435), or every flu season (RR: 0.263, 99% CI: 0.257, 0.268) when compared to those who never receive the vaccine. Risk ratios of annual seasonal influenza vaccination among children followed a similar pattern. A lower likelihood of non-vaccination was also observed among children with parents who were not reluctant or hesitant to vaccinate their child (RR: 0.444, 99% CI: 0.440, 0.448) or themselves (RR: 0.493, 99% CI: 0.488, 0.497). Children were more likely to be unvaccinated if they did not have asthma (RR: 1.162, 99% CI: 1.151, 1.173) or if they received only some (RR: 1.427, 99% CI: 1.407, 1.447) or none (RR: 1.735, 99% CI: 1.715, 1.755) of their childhood routine vaccinations. Parental intentions to vaccinate their child were also notably linked to an increased likelihood of the child being unvaccinated, particularly among those who definitely or probably won’t vaccinate their child next season in 2023–2024 (RR: 3.135, 99% CI: 3.102, 3.169), compared to those who definitely or probably will. Further, parents of lower household incomes (of $40,000 and less), when compared to household incomes of $150,000 or more, have a greater likelihood of having unvaccinated children (RR: 1.428, 99% CI: 1.400, 1.456), as are parents who are unvaccinated against seasonal influenza themselves (RR: 4.549, 99% CI: 4.480, 4.619).

**Figure 2:**
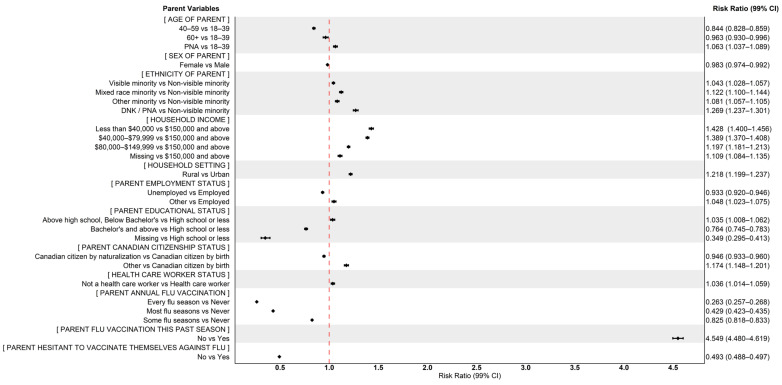
Forest plot of adjusted odds ratios for seasonal influenza non-vaccination among children and adolescents with chronic health conditions (Parent Variables).

**Figure 3:**
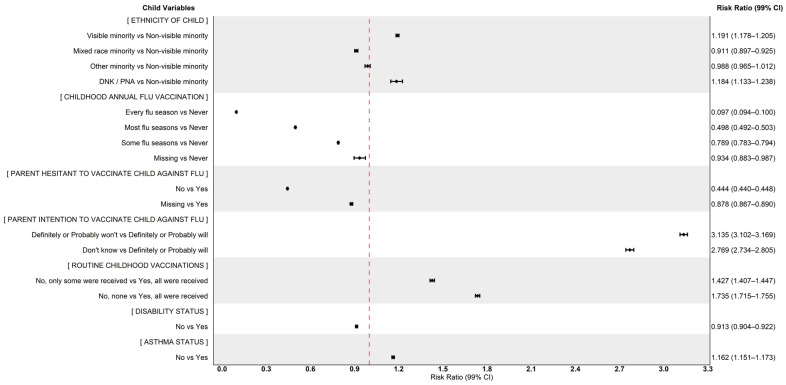
Forest plot of adjusted odds ratios for seasonal influenza non-vaccination among children and adolescents with chronic health conditions (Child Variables).

Figure 2 and Figure 3 plot age and sex (at birth) adjusted quasi-Poisson risk ratios (rounded to three significant decimals) for parent and child variables of interest.

## 4. Discussion

The seasonal influenza vaccine can protect youth with CHCs who are at increased risk of severe disease, complications, or hospitalization if infected. While there are studies documenting higher rates of flu vaccination coverage among children and adults with CHCs than among their healthy counterparts [30,31], this study found that over half (55%) of children 6 months to 17 years old with CHCs in Canada did not receive the seasonal influenza vaccine during the 2022–2023 season. However, according to published results from the 2022 Childhood COVID-19 Immunization Coverage Survey, 64% of Canadian youth with CHCs were unvaccinated against seasonal influenza during the previous 2021–2022 season, which suggests a marginal increase in vaccine coverage for this population took place between seasons [32]. Despite this subtle improvement coverage rates remain suboptimal, underscoring the need for sustained policy attention and targeted public health investment in seasonal influenza vaccination initiatives for high-risk populations such as youth with CHCs. Targeted outreach through primary care and school-based vaccination initiatives are a couple policy-based initiatives that can translate clinical recommendations into measurement improvements in vaccine coverage among this population.

In this study, children with CHCs were more likely to be unvaccinated against seasonal influenza during the 2022–2023 season if their parent was also unvaccinated against influenza that season. An Israeli study highlighted a similar finding among parents of children with cardiac disease, noting significantly greater odds of seasonal influenza vaccination for their children if they were vaccinated against seasonal influenza themselves [33]. Additionally, they found that children had significantly greater odds of vaccination when they were vaccinated annually, which also aligns well with findings from this study. This trend in annual vaccination extends to parents, given that annual parental vaccination was shown in this study to be strongly associated with lower rates of non-vaccination among their children with CHCs. These results were corroborated by an Australian cross-sectional study that investigated seasonal influenza vaccination uptake among children with CHCs and found that parents who received an annual vaccine had 11 times the odds of having a vaccinated child than those who did not receive the vaccine annually [34]. These studies, along with this study, suggest parental vaccination and annual seasonal influenza vaccination of both parents and youth are strong indicators of future vaccination behaviour within the household.

In fact, annual seasonal influenza vaccination is one of the most effective strategies for preventing infection and development of severe outcomes, given that each year vaccines are tailored to target predominant strains of circulating seasonal influenza virus [35]. Without this knowledge or understanding of vaccines, parents may not feel adequately informed or prepared to vaccinate themselves or their children. For instance, when Tuckerman et al. surveyed 539 Australian parents, they found that only 33% were aware that all children under the age of 5 should receive an annual seasonal influenza vaccine and 52% were aware of this recommendation for children with special risk medical conditions [36]. Despite poor vaccine awareness among some parents, vaccine recommendations from their general practitioner had positively influenced their decision to vaccinate their child in the future [36]. To investigate parental awareness further, Awad et al. conducted a study examining the effect vaccine awareness campaigns may have on parental perspectives towards seasonal influenza vaccines. They demonstrated that educational efforts to debunk vaccine myths and increase vaccine awareness positively shifted attitudes among 29% of parents who previously refused seasonal influenza vaccination for their children [37]. They also noted a parental preference to receive recommendations and information on seasonal influenza vaccines from doctors practicing in hospitals and clinics. Other studies further corroborate these findings [38,39], suggesting health care providers are a trusted source of information in the medical community, and thus have a unique responsibility to inform and remind parents on the importance of seasonal influenza vaccines. They should seize every opportunity to recommend an annual seasonal influenza vaccine to parents of young children, especially those with CHCs, address parental concerns regarding vaccination, and share educational resources on vaccination with parents when appropriate. It should be noted that while this study did not analyze data on provider-initiated vaccine recommendations, the associations described in the literature suggest that provider recommendations are a meaningful driver of parental vaccination decisions and should be further explored in research focused on children with CHCs.

Findings from this study also indicated a greater likelihood of non-vaccination among youth whose parents were reluctant or hesitant to vaccinate themselves or their child this 2022–2023 season, or who had no intention to vaccinate their child next season. This finding is consistent with several studies investigating the role vaccine hesitancy can play on present and future decisions to vaccinate [40,41]. The World Health Organization (WHO) Strategic Advisory Group of Experts on Immunization (SAGE) Working Group on Vaccine Hesitancy has defined vaccine hesitancy as a “delay in acceptance or refusal of vaccination despite availability of vaccination services” [42]. In addition, they developed the “3 Cs” model which focuses on three key factors of vaccine hesitancy: complacency, convenience, and confidence. In this study, parents who were vaccine hesitant or reluctant to vaccinate their child frequently cited concerns about the effectiveness and safety of the seasonal influenza vaccine. This finding suggests there is poor vaccine confidence (i.e., level of trust in vaccines) within this group. There are also a quarter of parents who are vaccine complacent and expressed a lack of concern regarding their child’s risk of infection and susceptibility to severe infection. Similar trends were shown among parents who refused to vaccinate their children, with vaccine complacency strongly exhibited by over half of parents who believed the seasonal influenza vaccine is unnecessary for their child with CHCs. The convenience component of this model points to structural barriers to vaccination, such as limited healthcare access, appointment availability and timing, timing constraints, and geographic distance from vaccination sites [34,43,44]. These widely documented convenience determinants of vaccine uptake may disproportionately affect lower-income families and those in rural settings. Although the present study did not directly measure structural barriers to vaccination, the associations observed between non-vaccination and lower household income and rural residence may partly reflect these upstream access-related constraints in addition to knowledge, attitude, and belief factors.

### Strengths and Limitations

To our knowledge, this study is the first of its kind to use a nationally representative Canadian sample of parents to determine factors associated with non-vaccination among children with CHCs. This study captures a unique snapshot of seasonal influenza vaccine coverage and parental beliefs among a vulnerable population of youth in Canada, of whom are specifically targeted for annual seasonal influenza vaccination. These findings can be compared to future research on this population to identify trends in vaccine coverage and beliefs over time. Parents were invited at random from all Canadian provinces and territories, thereby mitigating the potential for selection bias or geographical bias in this study. Further, sampling weights were developed using the 2021 Canadian census to ensure that weighted estimates accurately reflected the demographic distribution of the Canadian population.

While these strengths give credence to study findings, there are also some limitations. Inherent to all surveys, non-response bias may restrict the generalizability of findings to the broader population; however, there is limited variation between the demographic and geographic characteristics of the CCICS 2023 respondents compared to the Canadian population characteristics [16]. Additionally, reliance on self-reported data may introduce social desirability bias or recall bias which could overestimate or underestimate vaccine coverage in this study population.

By employing a cross-sectional design, vaccine coverage rates and perspectives were captured at a single moment in time and may have since evolved. The study results reflect seasonal influenza vaccination behaviours and attitudes from the 2022–2023 influenza season, during a period of heightened public divide in attitudes towards vaccines following the COVID-19 pandemic. However, polarizing perceptions towards vaccines persist [45], and seasonal influenza non-vaccination among youth with CHCs remains an important public health concern, with coverage rates declining in recent years [46].

Finally, removing cases with invalid or missing data when creating descriptive and analytic samples may have introduced bias into the study. Table A1 compares the final analytic sample (n = 1135) to those excluded due to missing data (n = 52). There were no significant differences between the two groups in child age, sex (at birth), child ethnicity, child asthma status, or parental employment status. However, there was a greater proportion of low household income parents (i.e., under $40,000) that were excluded from the final analytic sample (13.0% in excluded sample vs. 9.1% in analytic sample) and a smaller proportion of high household income parents that were excluded from the final analytic sample (24.5% in excluded sample vs. 32.9% in analytic sample), therefore findings may need to be interpreted with caution.

## 5. Conclusions

Despite the widespread availability of publicly funded seasonal influenza vaccines in Canada, along with strong recommendations for those at high-risk, this study found that seasonal influenza vaccine coverage among youth with CHCs was suboptimal and revealed several socio-demographic and vaccine-related factors associated with not receiving the vaccine during the 2022–2023 influenza season. Study results largely corroborate findings published in previous studies on this topic; however, the association between seasonal influenza vaccination status and household setting (or child ethnicity) among children with CHCs merits further investigation.

To increase parental awareness and receptivity towards seasonal influenza vaccines, efforts to develop and enhance messaging targeting parental gaps in vaccine knowledge is warranted. As trusted sources of information, health care providers can also directly address parental concerns towards seasonal influenza vaccines and provide resources for future reference. Furthermore, this study identified families of lower household incomes, those residing in rural settings, and children with an inconsistent seasonal influenza vaccination history as likely to be unvaccinated. By strategically enhancing and targeting public health initiatives for these subgroups, with community or mobile vaccination programmes, accessible multilingual messaging on seasonal influenza vaccines (via public transportation posters, webpages, community leaders, health care providers), and reminder-based interventions, vaccine uptake and annual adherence may improve. Forthcoming studies could expand on this work by evaluating public health interventions geared towards increasing vaccine uptake among children with CHCs.

## Figures and Tables

**Table 1:** Sociodemographic characteristics of parents of children 6 months–17 years with chronic health conditions, by seasonal influenza vaccination status, Canada, Childhood COVID-19 Immunization Coverage Survey, 2023.

Sociodemographic Factors	Child Vaccinated (n = 538)		Child Unvaccinated (n = 649)		*p*-Value ^b^
	Unweighted n	% (95% CI) ^a^	Unweighted n	% (95% CI) ^a^	
**Age of parent (in years)**					
18 to 39	148	27.2 (26.6, 27.9)	189	28.2 (27.7, 28.7)	0.21
40 to 59	366	68.4 (67.8, 69.0)	416	65.4 (65.0, 65.9)	
60 and older	8	1.3 (1.2, 1.4)	10	1.6 (1.6, 1.7)	
PNA	16	3.0 (2.9, 3.2)	34	4.8 (4.6, 4.9)	
**Sex of parent assigned at birth**					
Male	168	29.8 (29.5, 30.1)	190	30.4 (30.2, 30.7)	0.49
Female	368	70.2 (69.9, 70.5)	454	69.6 (69.3, 69.8)	
**Ethnicity of parent** ^c^					
Non-visible minority	428	78.7 (78.4, 78.9)	500	76.0 (75.8, 76.2)	0.40
Visible minority	77	14.8 (14.5, 15.0)	90	14.2 (14.1, 14.4)	
Mixed race	15	3.2 (3.1, 3.3)	27	4.5 (4.4, 4.6)	
Other	8	1.6 (1.5, 1.7)	11	2.0 (1.9, 2.1)	
DNK/PNA	10	1.8 (1.7, 1.8)	21	3.3 (3.2, 3.4)	
**Household income**					
Less than 40,000	33	6.7 (6.4, 6.9)	82	11.3 (11.1, 11.5)	<0.05
40,000 to 79,999	80	14.2 (13.9, 14.4)	138	20.7 (20.5, 20.9)	
80,000 to 149,999	182	33.0 (32.7, 33.3)	213	32.6 (32.4, 32.8)	
150,000 or more	202	38.3 (38.1, 38.6)	167	28.0 (27.8, 28.3)	
Missing	41	7.8 (7.6, 8.0)	49	7.4 (7.3, 7.6)	
**Household setting** ^d^					
Urban	455	88.7 (88.3, 89.0)	509	83.7 (83.5, 83.9)	<0.05
Rural	78	11.3 (11.0, 11.7)	131	16.3 (16.1, 16.5)	
**Parent Employment status**					
Employed	464	86.0 (85.8, 86.3)	550	86.9 (86.7, 87.0)	0.83
Unemployed	57	11.7 (11.5, 11.9)	75	10.6 (10.5, 10.8)	
Other	13	2.3 (2.2, 2.4)	17	2.5 (2.4, 2.6)	
**Parent Educational Level**					
High school or less	43	6.6 (6.3, 6.8)	67	9.0 (8.9, 9.2)	<0.05
Above high school, Below Bachelor’s	159	28.4 (28.2, 28.7)	281	42.7 (42.5, 42.9)	
Bachelor’s and above	328	63.2 (62.9, 63.5)	297	47.6 (47.3, 47.8)	
Missing	8	1.8 (1.7, 1.9)	NR	NR	
**Parent Canadian Citizenship status**					
Canadian Citizen by birth	441	80.8 (80.6, 81.1)	533	81.3 (81.1, 81.5)	0.74
Canadian Citizen by naturalization	76	16.4 (16.1, 16.6)	83	14.4 (14.2, 14.6)	
Other	21	2.8 (2.7, 2.9)	29	4.3 (4.2, 4.4)	
**Years parent living in Canada (Newcomer status)** ^g^					
Newcomer (≤5 years)	6	4.2 (3.9, 4.5)	12	10.6 (10.2, 10.9)	0.24
Long term resident (>6 years)	87	95.8 (95.5, 96.1)	95	89.4 (89.1, 89.8)	
**Health care worker status** ^f^					
Yes	108	18.0 (17.6, 18.4)	103	16.2 (16, 16.4)	0.06
No	430	82.0 (81.6, 82.4)	546	83.8 (83.6, 84)	
**Parent annual flu vaccination**					
Every flu season	259	47.1 (46.9, 47.4)	72	11.7 (11.5, 11.8)	<0.05
Most flu seasons	178	33.7 (33.4, 34.0)	121	17.8 (17.6, 18.0)	
Some flu seasons	71	14.2 (14.0, 14.4)	215	32.4 (32.2, 32.7)	
Never	29	4.9 (4.7, 5.2)	237	38.1 (37.8, 38.4)	
**Parent flu vaccination this past flu season** ^e^					
Yes	480	91.0 (90.7, 91.3)	112	17.9 (17.7, 18.1)	<0.05
No	55	9.0 (8.7, 9.3)	532	82.1 (81.9, 82.3)	
**Parent reluctant or hesitant to vaccinate themselves against flu this flu season**					
Yes	27	4.4 (4.3, 4.5)	249	38.8 (38.6, 39.0)	<0.05
No	507	95.6 (95.5, 95.7)	389	61.2 (61, 61.4)	

**Acronyms**: CI: Wald Confidence Interval; DNK: Don’t Know; PNA: Prefer Not to Answer. **Note:** Unweighted sample counts do not sum to total sample count (n = 1187) due to missing values for some sociodemographic factors. ^a^ Weighted percentages. ^b^ Pearson’s Chi-Square Test *p*-value using unweighted sample. ^c^ Non-visible minority represents White European descent; Visible minority represents all ethnicities other than White European descent, Mixed Race represents respondents who selected more than one ethnicity, Other represents respondents who provided their own response when specifying ethnicity. ^d^ This survey considered an urban area to be a city or village with a population of 1000 people or more, whereas a rural area is any area of lower population. ^e^ “Past season” refers to between September 2022 and March 2023. ^f^ This survey defined a “Health care worker” as having worked or volunteered as a health care or laboratory worker (excludes emergency service workers). ^g^ Excluding parents who are Canadian Citizens by birth.

**Table 2:** Sociodemographic characteristics of children 6 months–17 years with chronic health conditions, by seasonal influenza vaccination status, Canada, Childhood COVID-19 Immunization Coverage Survey, 2023.

Sociodemographic Factors	Child Vaccinated (n = 538)		Child Unvaccinated (n = 649)		*p*-Value ^c^
	Unweighted n	% (95% CI) ^a^	Unweighted n	% (95% CI) ^a^	
**Age of child (in years)**					
6 months–4 years	120	22.2 (21.4, 22.9)	98	14.8 (14.2, 15.5)	<0.05
5–11	206	41.6 (40.5, 42.7)	246	42.1 (41.0, 43.1)	
12–17	212	36.3 (35.3, 37.3)	305	43.1 (42.0, 44.1)	
**Sex of child assigned at birth**					
Male	321	59.2 (58.2, 60.1)	378	56.9 (55.9, 57.8)	0.62
Female	217	40.8 (39.9, 41.8)	271	43.1 (42.2, 44.1)	
**Ethnicity of child** ^b,d^					
Non-visible minority	387	70.4 (70.1, 70.7)	448	68.3 (68.0, 68.6)	0.27
Visible minority	55	9.8 (9.6, 10.0)	88	13.8 (13.6, 13.9)	
Mixed race	71	14.8 (14.6, 15.1)	74	11.8 (11.6, 11.9)	
Other	10	2.3 (2.2, 2.4)	13	2.3 (2.3, 2.4)	
**Disability status of child** ^e^					
Yes	144	28.1 (27.8, 28.3)	214	32.9 (32.6, 33.1)	<0.05
No	384	71.9 (71.7, 72.2)	422	67.1 (66.9, 67.4)	
**Asthma status of child**					
Yes	338	64.1 (63.8, 64.4)	356	55.2 (54.9, 55.4)	<0.05
No	200	35.9 (35.6, 36.2)	293	44.8 (44.6, 45.1)	
**Childhood annual flu vaccination**					
Every flu season	358	65.3 (65.0, 65.6)	37	5.5 (5.4, 5.6)	<0.05
Most flu seasons	105	20.3 (20.0, 20.6)	92	14.2 (14.0, 14.3)	
Some flu seasons	53	10.6 (10.4, 10.8)	181	26.3 (26.1, 26.5)	
Never	17	3.0 (2.9, 3.1)	309	49.1 (48.8, 49.3)	
Missing	5	0.8 (0.6, 1.0)	30	4.9 (4.8, 5.0)	
**Parent reluctant or hesitant to vaccinate child against flu this flu season**					
Yes	17	2.6 (2.3, 2.8)	262	41.6 (41.3, 41.9)	<0.05
No	518	96.7 (96.4, 96.9)	367	55.1 (54.9, 55.4)	
Missing	NR	NR	20	3.3 (3.2, 3.3)	
**Parent intention to vaccinate child against flu next flu season**					
Definitely or Probably will	519	96.7 (96.5, 97.0)	234	34.5 (34.3, 34.7)	<0.05
Definitely or Probably won’t	7	1.3 (1.1, 1.5)	349	54.6 (54.4, 54.8)	
Don’t know	12	2.0 (1.9, 2.1)	65	10.9 (10.8, 11.0)	
**Routine childhood vaccinations**					
Yes, all were received	514	95.7 (95.6, 95.8)	558	86.2 (86.1, 86.4)	<0.05
No, only some were received	20	3.6 (3.5, 3.7)	67	10.2 (10.1, 10.4)	
No, none were received	NR	NR	20	3.5 (3.5, 3.6)	

**Acronyms**: CI: Wald Confidence Interval; NR: Not reportable due to small cell counts (<5) or for confidentiality purposes. **Note:** Unweighted sample counts do not sum to total sample count (n = 1187) due to missing values for some sociodemographic factors. ^a^ Weighted percentages. ^b^ Values for “Don’t know” and/or “Prefer Not to Answer” are not presented in this table for confidentiality purposes. ^c^ Pearson’s Chi-Square Test *p*-value using unweighted sample. ^d^ Non-visible minority represents White European descent; Visible minority represents all ethnicities other than White European descent, Mixed Race represents respondents who selected more than one ethnicity, Other represents respondents who provided their own response when specifying ethnicity. ^e^ Disability represents “a long-term or recurring impairment (such as vision, hearing, mobility, flexibility, dexterity, pain, learning, developmental, memory or mental health-related) which limits their daily activities inside or outside the home (such as at school, work, or in the community in general).” [Excerpt from survey text].

**Table 3:** Top seven reasons for non-vaccination against seasonal influenza reported by parents who were hesitant or reluctant to vaccinate their child with at least one chronic health condition (n = 262), Canada, Childhood COVID-19 Immunization Coverage Survey, 2023.

	Number (n)	Percent (%) ^b^
**Reasons for non-vaccination against seasonal influenza among hesitant or reluctant parents (n = 262) ^a^**		
I was concerned about the effectiveness	103	39.1%
I had concerns about the safety	77	27.5%
My child is not at risk of getting the flu or at risk of severe infection	68	25.3%
My child had a bad experience with previous vaccines	37	12.3%
Other	36	14.0%
My child fears needles	28	9.9%
Religious or philosophical reasons	15	4.7%

^a^ These reasons are not mutually exclusive. Participants may select more than one reason. ^b^ Weighted percentages.

**Table 4:** Top seven reasons for non-vaccination against seasonal influenza reported by parents who refused to vaccinate their child with at least one chronic health condition (n = 229), Canada, Childhood COVID-19 Immunization Coverage Survey, 2023.

	Number (n)	Percent (%) ^b^
**Reasons for non-vaccination against seasonal influenza among parents who refused vaccination (n = 229)** ^a^		
Did not consider it necessary for my child	130	56.1%
Concerns about the safety/side effects of the flu vaccine	84	35.8%
The flu vaccine does not work	59	24.9%
Concerns about receiving a COVID-19 and flu vaccine at the same time	40	17.2%
Other	34	14.9%
My child is not at risk of getting the flu	22	9.2%
Religious or philosophical reasons	13	5.0%

^a^ These reasons are not mutually exclusive. Participants may select more than one reason. ^b^ Weighted percentages.

**Table 5:** Unadjusted and adjusted risk ratios for the association between sociodemographic characteristics and seasonal influenza non-vaccination among children and adolescents 6 months-17 years with chronic health conditions, Canada, Childhood COVID-19 Immunization Coverage Survey, 2023.

Sociodemographic Factors	Unadjusted RR ^b^ (99% CI)	Adjusted RR ^b^ for Child Age and Sex (99% CI)	Fully Adjusted RR ^c^ (99% CI)
**Age of parent (in years)**			
18 to 39	Reference	Reference	--
40 to 59	0.975 (0.959, 0.990)	0.844 (0.828, 0.859)	--
60 and older	1.106 (1.070, 1.143)	0.963 (0.930, 0.996)	--
PNA	1.217 (1.190, 1.245)	1.063 (1.037, 1.089)	--
**Sex of parent assigned at birth**			
Male	Reference	Reference	--
Female	0.977 (0.967, 0.987)	0.983 (0.974, 0.992)	--
**Ethnicity of parent ^a^**			
Non-visible minority	Reference	Reference	--
Visible minority	1.030 (1.015, 1.045)	1.043 (1.028, 1.057)	--
Mixed race	1.111 (1.089, 1.133)	1.122 (1.100, 1.144)	--
Other	1.124 (1.099, 1.149)	1.081 (1.057, 1.105)	--
DNK/PNA	1.278 (1.244, 1.312)	1.269 (1.237, 1.301)	--
**Household income**			
Less than 40,000	1.415 (1.386, 1.444)	1.428 (1.400, 1.456)	1.034 (1.012, 1.057)
40,000 to 79,999	1.372 (1.353, 1.392)	1.389 (1.370, 1.408)	0.992 (0.981, 1.003)
80,000 to 149,999	1.176 (1.160, 1.192)	1.197 (1.181, 1.213)	1.041 (1.032, 1.051)
150,000 or more	Reference	Reference	Reference
Missing	1.113 (1.088, 1.138)	1.109 (1.084, 1.135)	1.004 (0.989, 1.018)
**Household setting** ^d^			
Urban	Reference	Reference	Reference
Rural	1.191 (1.173, 1.210)	1.218 (1.199, 1.237)	0.961 (0.948, 0.974)
**Parent Employment status**			
Employed	Reference	Reference	Reference
Unemployed	0.938 (0.924, 0.951)	0.933 (0.920, 0.946)	0.913 (0.903, 0.922)
Other	1.076 (1.050, 1.104)	1.048 (1.023, 1.075)	0.902 (0.881, 0.924)
**Parent Educational status**			
High school or less	Reference	Reference	Reference
Above high school, Below Bachelor’s	1.044 (1.015, 1.074)	1.035 (1.008, 1.062)	1.047 (1.017, 1.077)
Bachelor’s and above	0.766 (0.747, 0.786)	0.764 (0.745, 0.783)	1.004 (0.975, 1.033)
Missing	0.330 (0.280, 0.389)	0.349 (0.295, 0.413)	0.688 (0.617, 0.768)
**Parent Canadian Citizenship status**			
Canadian Citizen by birth	Reference	Reference	Reference
Canadian Citizen by naturalization	0.947 (0.934, 0.961)	0.946 (0.933, 0.960)	0.912 (0.904, 0.921)
Other	1.161 (1.135, 1.188)	1.174 (1.148, 1.201)	1.003 (0.985, 1.021)
**Health care worker status** ^e^			
Yes	Reference	Reference	Reference
No	1.043 (1.020, 1.066)	1.036 (1.014, 1.059)	0.963 (0.941, 0.985)
**Parent annual flu vaccination**			
Every flu season	0.264 (0.259, 0.270)	0.263 (0.257, 0.268)	--
Most flu seasons	0.433 (0.426, 0.439)	0.429 (0.423, 0.435)	--
Some flu seasons	0.839 (0.830, 0.847)	0.825 (0.818, 0.833)	--
Never	Reference	Reference	--
**Parent flu vaccination this past season** ^f^			
Yes	Reference	Reference	Reference
No	4.574 (4.505, 4.644)	4.549 (4.480, 4.619)	2.310 (2.279, 2.341)
**Parent reluctant or hesitant to vaccinate themselves against flu**			
Yes	Reference	Reference	--
No	0.488 (0.483, 0.492)	0.493 (0.488, 0.497)	--
**Age of child (in years)**			
6 months–4 years	Reference	--	Reference
5–11	1.216 (1.195, 1.238)	--	1.036 (1.025, 1.048)
12–17	1.319 (1.299, 1.339)	--	1.050 (1.038, 1.062)
**Sex of child**			
Male	Reference	--	Reference
Female	1.041 (1.028, 1.054)	--	1.020 (1.014, 1.027)
**Ethnicity of child** ^a^			
Non-visible minority	Reference	Reference	Reference
Visible minority	1.195 (1.181, 1.209)	1.191 (1.178, 1.205)	1.068 (1.053, 1.084)
Mixed race	0.907 (0.893, 0.921)	0.911 (0.897, 0.925)	1.043 (1.030, 1.056)
Other	0.991 (0.966, 1.016)	0.988 (0.965, 1.012)	1.007 (0.991, 1.024)
DNK/PNA	1.195 (1.141, 1.252)	1.184 (1.133, 1.238)	1.080 (1.012, 1.153)
**Childhood annual flu vaccination**			
Every flu season	0.096 (0.093, 0.099)	0.097 (0.094, 0.100)	0.213 (0.206, 0.221)
Most flu seasons	0.501 (0.496, 0.507)	0.498 (0.492, 0.503)	0.840 (0.829, 0.851)
Some flu seasons	0.793 (0.788, 0.798)	0.789 (0.783, 0.794)	0.976 (0.969, 0.983)
Never	Reference	Reference	Reference
Missing	0.941 (0.890, 0.994)	0.934 (0.883, 0.987)	1.000 (0.941, 1.062)
**Parent reluctant or hesitant to vaccinate child against flu this flu season**			
Yes	Reference	Reference	Reference
No	0.442 (0.438, 0.446)	0.444 (0.440, 0.448)	0.909 (0.903, 0.916)
Missing	0.875 (0.861, 0.889)	0.878 (0.867, 0.890)	0.926 (0.914, 0.937)
**Parent intention to vaccinate child against flu next flu season**			
Definitely or Probably will	Reference	Reference	Reference
Definitely or Probably won’t	3.155 (3.121, 3.189)	3.135 (3.102, 3.169)	1.159 (1.146, 1.172)
Don’t know	2.781 (2.745, 2.817)	2.769 (2.734, 2.805)	1.163 (1.149, 1.177)
**Routine childhood vaccinations**			
Yes, all were received	Reference	Reference	--
No, only some were received	1.437 (1.418, 1.457)	1.427 (1.407, 1.447)	--
No, none	1.702 (1.684, 1.720)	1.735 (1.715, 1.755)	--
**Disability status of child** ^g^			
Yes	Reference	Reference	Reference
No	0.899 (0.890, 0.908)	0.913 (0.904, 0.922)	1.007 (0.997, 1.017)
**Asthma (or chronic lung disease) status of child**			
Yes	Reference	Reference	Reference
No	1.184 (1.173, 1.196)	1.162 (1.151, 1.173)	1.019 (1.012, 1.025)

**Acronyms**: CI: Wald Confidence Interval; DNK: Don’t Know; RR: Risk Ratio; PNA: Prefer Not to Answer. ^a^ Non-visible minority represents White European descent; Visible minority represents all ethnicities other than White European descent, Mixed Race represents respondents who selected more than one ethnicity, Other represents respondents who provided their own response when specifying ethnicity. ^b^ Sample size for this age and sex adjusted quasi-Poisson regression is n = 1135. ^c^ Sample size for this multivariate quasi-Poisson regression is n = 1135; model was adjusted for all variables whose risk ratios are presented in this column. ^d^ This survey considered an urban area to be a city or village with a population of 1000 people or more, whereas a rural area is any area of lower population. ^e^ This survey defined a “Health care worker” as having worked or volunteered as a health care or laboratory worker (excludes emergency service workers). ^f^ “This season” refers to between September 2022 and March 2023. ^g^ Disability represents “a long-term or recurring impairment (such as vision, hearing, mobility, flexibility, dexterity, pain, learning, developmental, memory or mental health-related) which limits their daily activities inside or outside the home (such as at school, work, or in the community in general).” [Excerpt from survey text].

## Data Availability

The authors do not have permission to share study data due to data sensitivity and confidentiality. Detailed results tables and the methodological report can be accessed through the Library and Archives Canada website at: Childhood COVID-19 Immunization Coverage Survey (CCICS), 2023: methodological report.

## Abbreviations

The following abbreviations are used in this manuscript:

CCICS	Childhood COVID-19 Immunization Coverage Survey
CHC	Chronic health conditions
CI	Confidence Interval
DNK	Do not know
NR	Not reportable
PNA	Prefer not to answer
ROC	Receiver Operating Characteristic
RR	Risk Ratio
SAGE	Strategic Advisory Group of Experts on Immunization
WHO	World Health Organization

## Appendix A

**Table A1:** Sociodemographic characteristics of analytic sample (for multivariate quasi-Poisson regression) and excluded sample, Canada, Childhood COVID-19 Immunization Coverage Survey, 2023.

Sociodemographic Characteristics	Analytic Sample (Multivariate Quasi-Poisson Regression) (n = 1135)		Excluded Sample ^b^ (Missing Data) (n = 52)		*p*-Value ^a^
	Unweighted n	Unweighted % (95% CI)	Unweighted n	Unweighted % (95% CI)	
**Age of child (in years)**					
6 months–4 years	209	18.2 (17.5, 18.9)	9	14.8 (14.0, 15.7)	0.94
5–11 years	431	40.4 (39.4, 41.4)	21	33.2 (32.0, 34.5)	
12–17 years	495	41.4 (40.4, 42.4)	22	51.9 (50.5, 53.4)	
**Sex of child assigned at birth**					
Male	665	57.8 (56.9, 58.8)	34	59.2 (57.9, 60.5)	0.33
Female	470	42.2 (41.2, 43.1)	18	40.8 (39.5, 42.1)	
**Ethnicity of child** ^c,d^					
Non-visible minority	801	69.4 (69.2, 69.7)	34	63.8 (62.9, 64.7)	0.72
Visible minority	136	11.8 (11.7, 11.9)	7	17.7 (17.1, 18.3)	
Mixed race	138	13.1 (13.0, 13.3)	7	12.1 (11.5, 12.6)	
Other	22	2.3 (2.2, 2.4)	NR	NR	
**Parent Employment Status**					
Employed	979	86.5 (86.3, 86.6)	35	87.9 (87.2, 88.6)	0.92
Unemployed	127	11.1 (11.0, 11.3)	5	9.4 (8.9, 9.9)	
Other	29	2.4 (2.3, 2.4)	NR	NR	
**Household income**					
Under $40,000	106	9.1 (8.9, 9.2)	9	13.0 (12.1, 14.0)	<0.05
$40,000 to $79,999	213	18.1 (18.0, 18.3)	5	11.4 (10.9, 11.9)	
$80,000 to $149,999	382	33.1 (32.9, 33.3)	13	26.2 (25.4, 27.1)	
$150,000 or above	355	32.9 (32.7, 33.1)	14	24.5 (23.7, 25.3)	
Missing	79	6.8 (6.7, 6.9)	11	24.8 (24.0, 25.6)	
**Asthma status of child**					
Yes	465	59.9 (59.6, 60.1)	23	53.1 (52.1, 54.1)	0.64
No	670	40.1 (39.9, 40.4)	29	46.9 (45.9, 47.9)	

**Acronyms**: CI: Wald Confidence Interval; NR: Not reportable due to small cell counts (<5) or for confidentiality purposes. **Note**: Unweighted sample counts do not necessarily sum to total sample count (n = 1135, n = 52) due to missing values for some sociodemographic factors. ^a^ Pearson’s Chi-Square Test *p*-value (using unweighted cell counts) was computed if all expected cell counts are greater than 5; else Fisher’s exact test *p*-value was computed if at least one expected cell count was less than 5. ^b^ Excluded Sample includes parents of youth and adolescents (6 months–17 years old), with chronic health conditions, and who had valid responses to study variables used to define analytic sample, but were excluded due to missing data for other sociodemographic variables of interest (e.g., education or employment). ^c^ Values for “Don’t know” and/or “Prefer Not to Answer” are not presented in this table for confidentiality purposes. ^d^ Non-visible minority represents White European descent; Visible minority represents all ethnicities other than White European descent, Mixed Race represents respondents who selected more than one ethnicity, Other represents respondents who provided their own response when specifying ethnicity.
